# Supplementary figures and images for: Chemokines act as phosphatidylserine-bound “find-me” signals in apoptotic cell clearance
Source: PLoS Biol. 2021 May 26;19(5):e3001259. doi: 10.1371/journal.pbio.3001259 (PMC8213124; doi:10.1371/journal.pbio.3001259)

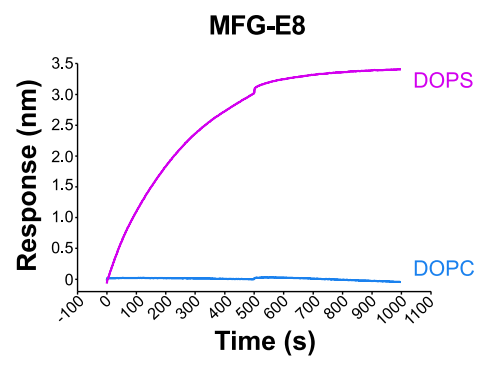

Supplement: S1 Fig — Binding of recombinant MFG-E8 (200 nM) to BLI biosensors immobilized with DOPC (blue sensorgram) or DOPS (pink sensorgram) liposomes. BLI, biolayer interferometry; MFG-E8, milk fat globule-epidermal growth factor 8; PS, phosphatidylserine. (PDF) [file pbio.3001259.s001.pdf]

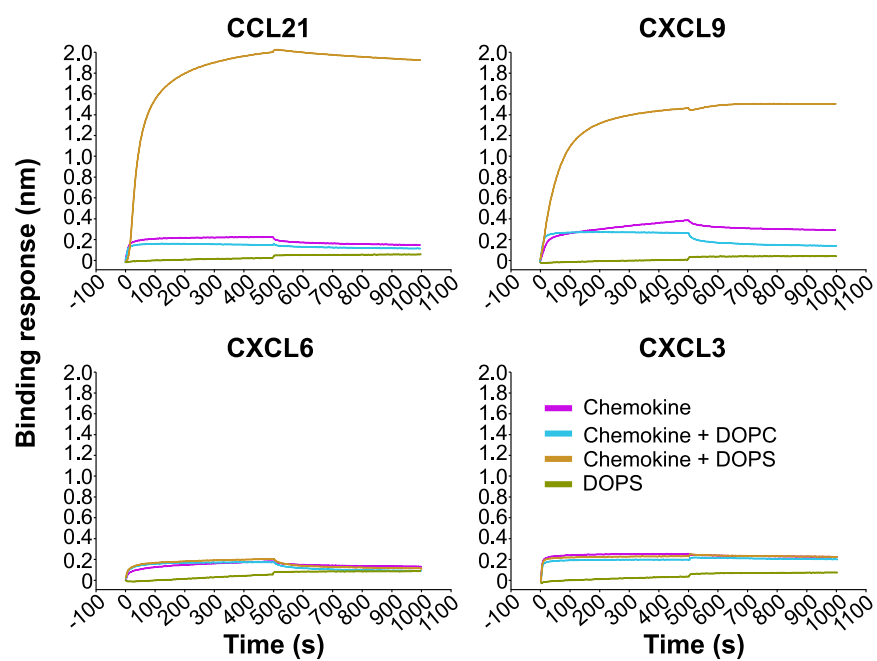

Supplement: S2 Fig — Primary antibodies used in ELISA or protein–lipid overlay assays to detect CCL21, CXCL9, CXCL6, or CXCL3 were immobilized onto BLI amine-reactive biosensors. BLI binding sensorgrams for the interaction of DOPS liposomes alone (green) and 400 nM of each chemokine alone (magenta) or preincubated with 0.5 mg/ml of DOPS (yellow) or DOPC (blue) liposomes are shown. Increase in the binding response in the presence of liposomes is indicative of the binding of a large analyte (chemokine–liposome complex). BLI, biolayer interferometry. (PDF) [file pbio.3001259.s002.pdf]

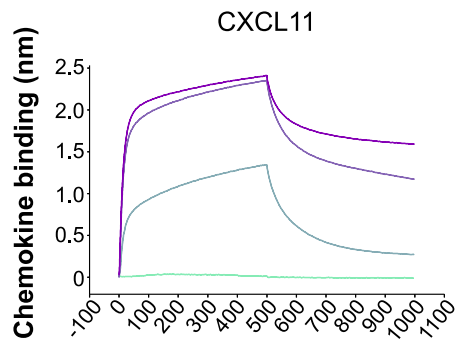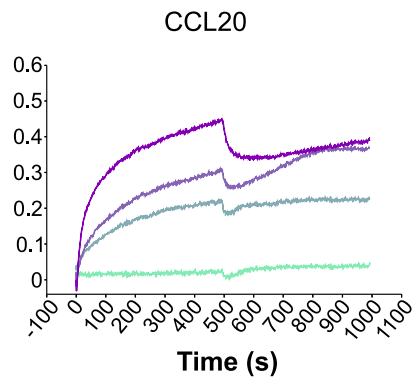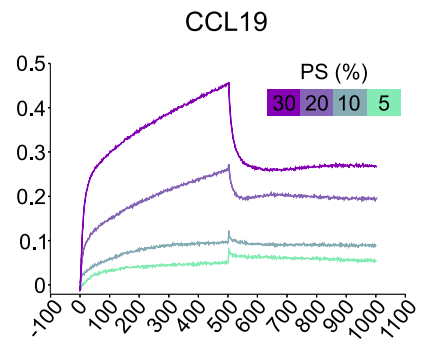

Supplement: S3 Fig — BLI experiments showing the binding of the indicated chemokines (500 nM) to DOPS liposomes containing decreasing amounts of PS (as indicated in the inset of the CCL19 graph). Binding to DOPC liposomes was subtracted from all binding curves. BLI, biolayer interferometry; PS, phosphatidylserine. (PDF) [file pbio.3001259.s003.pdf]

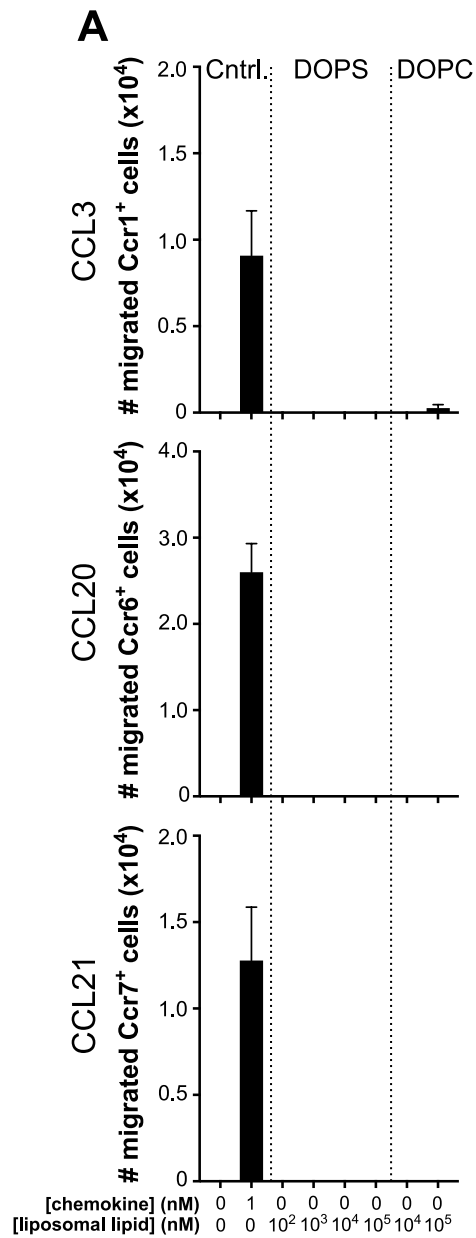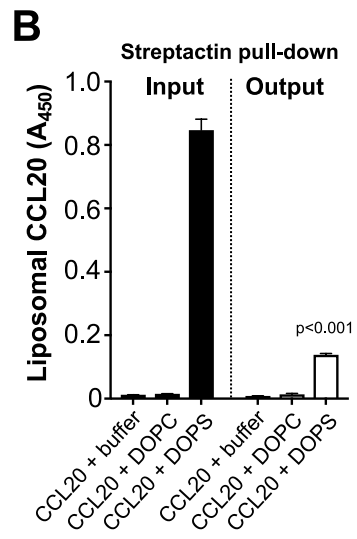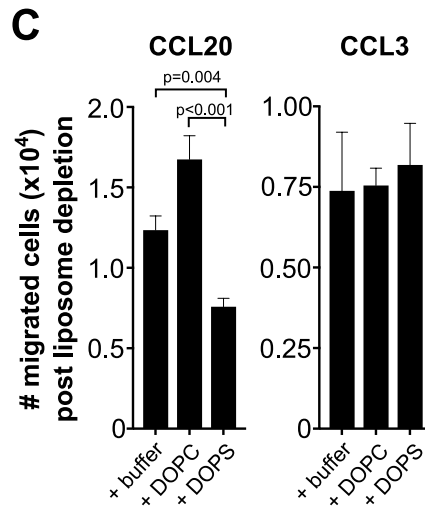

Supplement: S4 Fig — (A) Liposomes do not induce chemotaxis in the absence of chemokine. Cell migration of Ccr1-, Ccr6-, and Ccr7-expressing L1.2 cells (y-axis) in the presence of the same concentrations (x-axis) of DOPS or DOPC liposomes used in Fig 3A but without chemokine was assayed in transwell plates for 3–4 hours at 37°C. Media alone (0:0, chemokine:lipid molar ratio) and 1 nM of the appropriate chemokine agonist (as indicated on the left side of each graph) in the absence of liposome (1:0, chemokine:lipid molar ratio) were included as negative and positive controls, respectively. Results from controls (Cntrl.) and cells stimulated with DOPS or DOPC liposomes are separated by vertical dashed lines and labeled above the top graph. Bars represent the mean ± SD of triplicate determinations from one experiment representative of 2 independent experiments. (B) Pull-down of CCL20–DOPS liposome complexes decreases their availability in solution. CCL20 (1 nM) was incubated with buffer or a 104-fold molar excess of DOPC or DOPS liposomes doped with a small amount of biotinylated DOPE. A total of 50 μl of the liposome suspension before (input) and after (output) pull-down with 30 μl of Strep-Tactin beads were analyzed in triplicate by ELISA in streptavidin-coated plates. Liposome-bound CCL20 was detected with a rabbit anti-CCL20 polyclonal Ab followed by an HRP-conjugated anti-rabbit antibody, and the A450 was determined after development with TMB One Component solution. Bars represent the mean ± SD of data from one experiment representative of 2 independent experiments. The p-value from a 2-tailed t test for the analysis of CCL20 + DOPS input vs. output is indicated. (C) Depletion of CCL20–DOPS liposome complexes by pull-down reduces cell migration. CCL3 or CCL20 (as indicated above each graph, 1 nM) were incubated with buffer or a 104-fold molar excess of DOPC or DOPS liposomes. Then, chemokine–liposome complexes were pulled down with Strep-Tactin-beads, and the chemokine activity remaini [file pbio.3001259.s004.pdf]

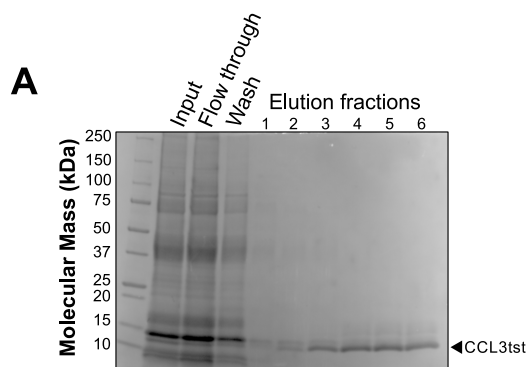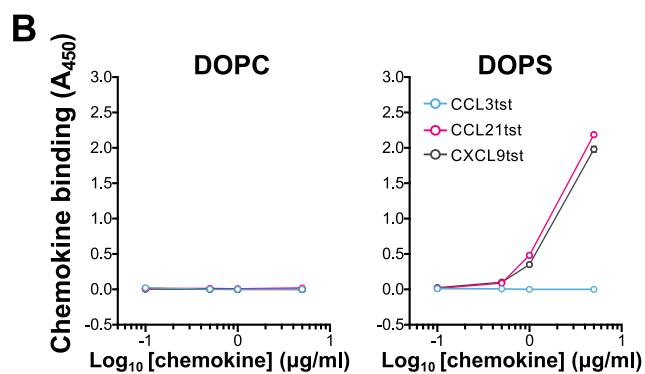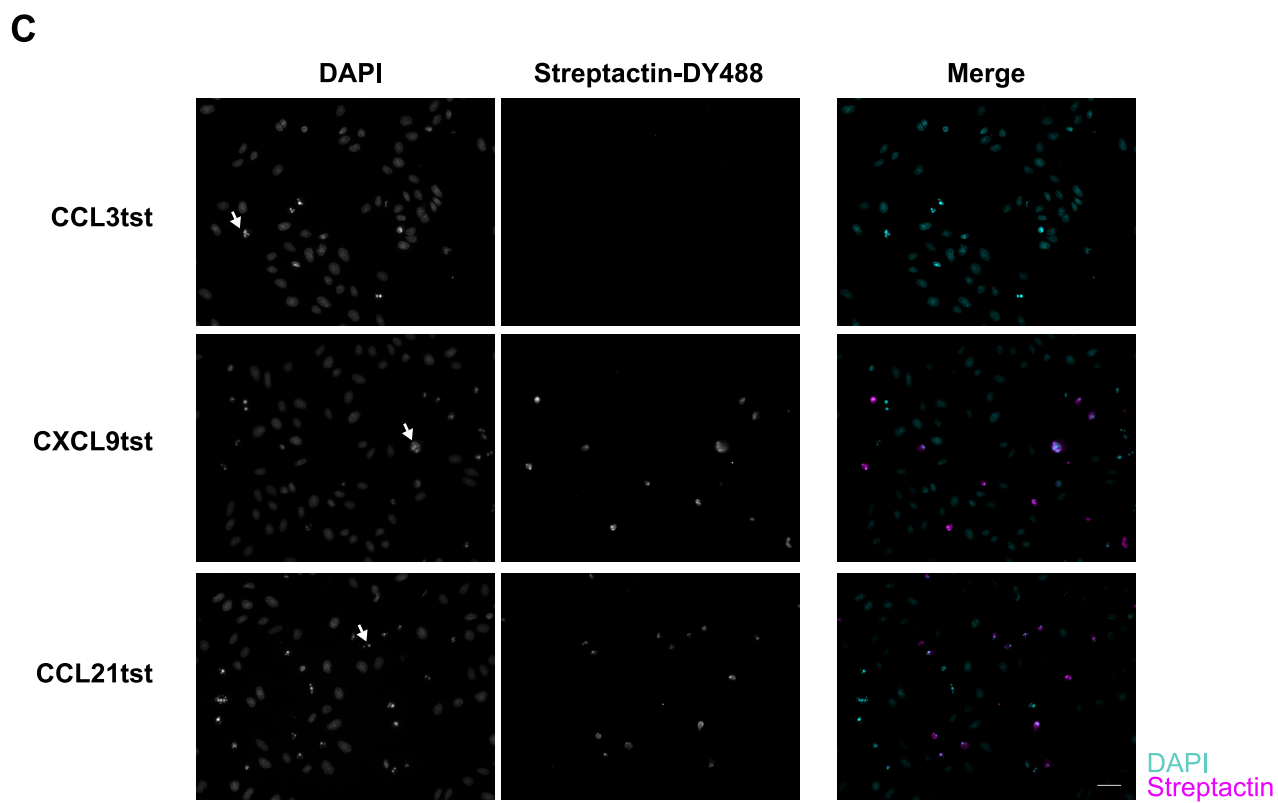

Supplement: S5 Fig — (A) Purification of recombinant CCL3 expressed in Expi293F cells. Coomassie-stained acrylamide gel showing the purification steps of human CCL3. Human CCL3, CXCL9, and CCL21 were tagged with a twin-strep tag (tst) at the carboxyl terminus and expressed in Expi293F cells. Recombinant proteins were purified from cell SNs by affinity chromatography using Strep-Tactin XT columns. (B) CXCL9tst and CCL21tst interact with PS-containing liposomes. The binding of in-house produced glycosylated recombinant chemokines to DOPC or DOPS liposomes was analyzed by ELISA. Increasing doses (x-axis) of the different chemokines (as indicated in the inset of the right panel) were incubated in wells immobilized with DOPC (left panel) or DOPS (right panel) liposomes. Wells were washed extensively with TBS, then bound chemokine was detected with an HRP-conjugated anti-tst mAb, and the A450 was determined after developing with TMB One Solution substrate. Data are the mean ± SD of triplicates from one experiment representative of 3 independent experiments. (C) PS-binding chemokines interact with the surface of dying CHO-745 cells. Immunofluorescence images show the binding of CCL3tst, CXCL9tst, and CCL21tst (as indicated on the left side of each row) to UV-irradiated CHO-745 cells. Cells cultured on coverslips were exposed to 100 mJ of UV light using a Stratalinker. Six hours after treatment, coverslips were incubated with 400 nM of each chemokine in AnV binding buffer. After washing, samples were stained with Strep-Tactin XT conjugated with DY-488, then fixed and mounted using Prolong Gold with DAPI. Epifluorescence images were acquired in a Zeiss Anxiovert 200M inverted microscope (200× magnification). DAPI (first column), Strep-Tactin staining (second column), and merge images (third column) are shown. White arrows in the DAPI panels point at examples of dying cells displaying fragmented and condensed nuclei. In the merge panels, DAPI and Strep-Tactin staining are shown in cyan and magent [file pbio.3001259.s005.pdf]

**A**

# CHO-745 cells

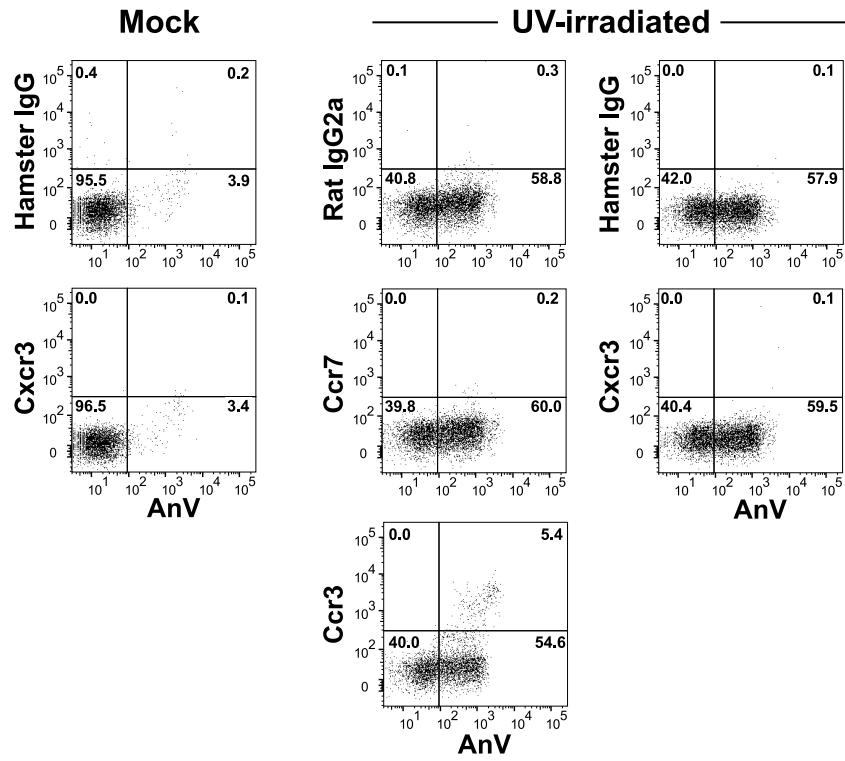

**B**

# Thymocytes

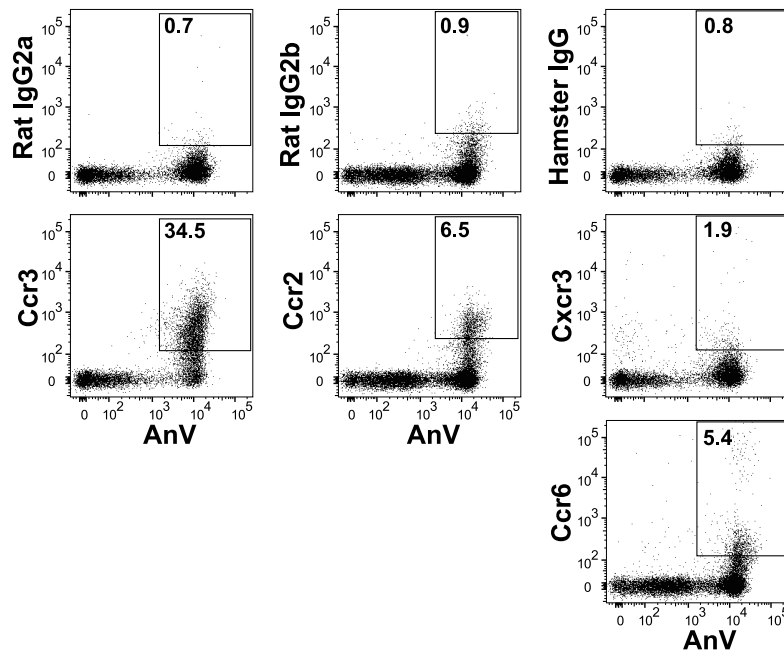

Supplement: S6 Fig — The expression of the cellular receptors for the chemokines included in the cell-binding assays shown in Fig 4 was analyzed by FACS in CHO-745 cells (A) and mouse thymocytes (B) as indicated above each panel. (A) Cxcr3, Ccr3, and Ccr7 are not expressed in live or apoptotic CHO-745 cells. Mock-treated or UV-irradiated CHO-745 cells (as indicated above the graph columns) were stained with AnV-APC and R-PE conjugated antibodies for the chemokine receptors Ccr3, Ccr7, and Cxcr3 (as indicated on the y-axis of the corresponding graphs). Chemokine receptor-AnV dot plots are shown. Dot plots for the staining with the pertinent isotype controls are shown above the corresponding columns. (B) Ccr3, and to a lower extent, Ccr2 and Ccr6, but not Cxcr3, are expressed specifically by apoptotic thymocytes. Freshly isolated mouse thymocytes were incubated with 1 μM DEX at 37°C. After 4 hours, thymocytes were stained with AnV-APC and PE-conjugated anti-Ccr2, anti-Ccr3, anti-Ccr6, or anti-Cxcr3 antibodies as indicated on the y-axis of the corresponding graphs. Chemokine receptor-AnV dot plots are shown below the corresponding isotype control for each anti-chemokine receptor antibody. In both panels A and B, numbers indicate the % of events in each gate. AnV, annexin V; DEX, dexamethasone; R-PE, R-Phycoerythrin; UV, ultraviolet. (PDF) [file pbio.3001259.s006.pdf]

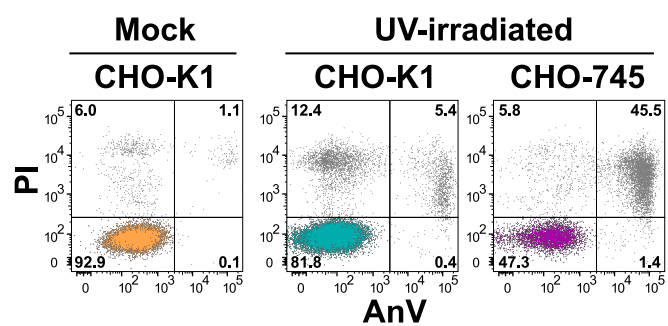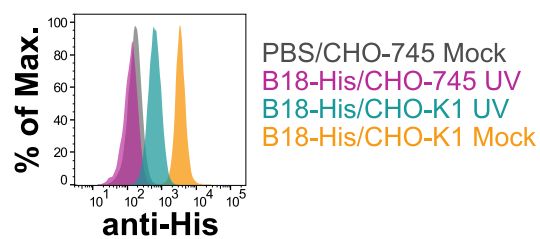

Supplement: S7 Fig — CHO-K1 (GAG competent) and CHO-745 (GAG deficient) cells were exposed to 100 mJ of UV-light. Six hours after irradiation, cells were collected, and the cell binding of the GAG-binding B18-His protein was analyzed by FACS with an anti-His mAb followed by an anti-mouse Alexa Fluor 488-conjugated antibody. Before the analysis, cells were stained with PI and APC-conjugated AnV. Top dot plots show the PI and AnV staining of mock- and UV-irradiated CHO-K1 or CHO-745 cells as indicated above each graph. Numbers correspond to % of events in each gate. Bottom histogram graph shows the binding of B18-His to AnV− PI− populations (color coded in the top dot plots) from mock- (orange) or UV-irradiated CHO-K1 (green) and CHO-745 cells (purple) as indicated in the legend on the right side of the graph. Staining of CHO-745 cells in the absence of B18-His (PBS, gray) is shown as reference. AnV, annexin V; PI, propidium iodide; UV, ultraviolet. (PDF) [file pbio.3001259.s007.pdf]

**A**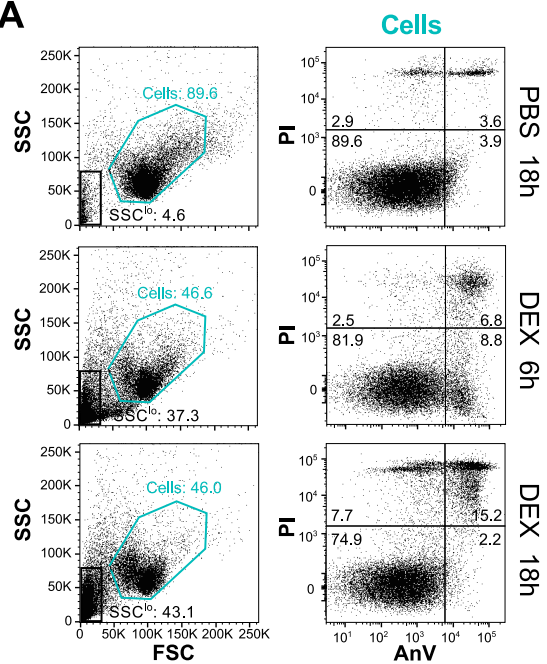**B**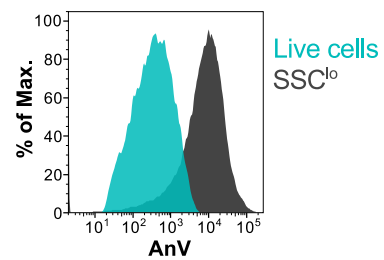

Supplement: S8 Fig — (A) C57BL/6j mice were i.p. injected with PBS or DEX, and thymocytes were isolated 6 hours or 18 hours after treatment (as indicated on the right side of each graph row) and stained with PI and APC-conjugated AnV. In the left column, SSC-FSC dot plots showing gates for the cells (Cells, blue) and apoptotic blebs (SSClo, black). In the right column, PI-AnV dot plots of the events from the “Cells” gate of each condition. Numbers indicate the % of events in each gate. (B) Histograms of the AnV staining of live cells (AnV− PI−) and apoptotic blebs (SSClo, black). AnV, annexin V; DEX, dexamethasone; FSC, forward scatter; i.p., intraperitoneal; PI, propidium iodide; PS, phosphatidylserine; SSC, side scatter. (PDF) [file pbio.3001259.s008.pdf]

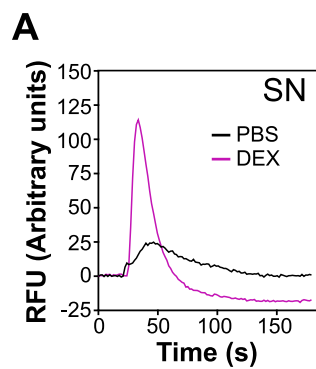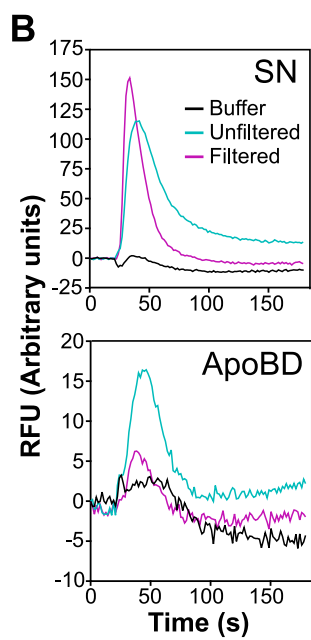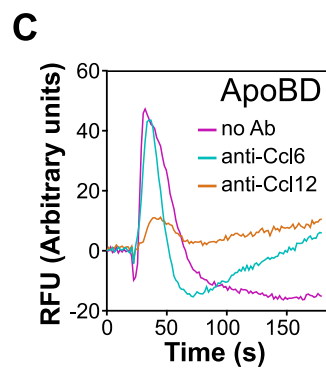

Supplement: S9 Fig — (A) DEX treatment is required for strong Ccr2 activation by SN fractions isolated from mouse thymus homogenates. Calcium flux of Ccr2-expressing L1.2 cells in response to thymic SN isolated from PBS- (black) or DEX-inoculated (pink) mice. (B) Calcium flux response for buffer alone (black) and for the mouse SN and ApoBD fractions previously filtered (pink) or not (blue) through a 0.2 μm filter. (C) Calcium flux response obtained with the ApoBD fraction preincubated or not (No Ab, pink) with 10 μg/ml of an anti-Ccl12 antibody (orange) or a control anti-Ccl6 antibody (blue). All SN and ApoBD fractions were isolated 18 hours after mouse treatment. Calcium recordings correspond to the mean of duplicates from one experiment representative of 2 independent experiments. The underlying numerical values for the panels displaying summary numerical data can be found in S1 Data. ApoBD, apoptotic body; DEX, dexamethasone; RFU, relative fluorescence units; SN, supernatant. (PDF) [file pbio.3001259.s009.pdf]

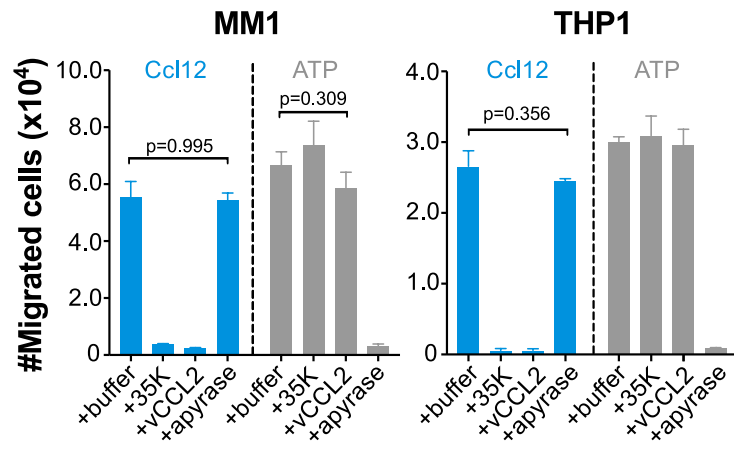

Supplement: S10 Fig — Chemotaxis assays proving the chemokine or ATP inhibitory specificity of 35K and vCCL2, or apyrase, respectively. Migration of MM1 and THP1 cells induced by Ccl12 (1 nM) or ATP (500 nM) preincubated with buffer, 200 nM of the chemokine inhibitors 35K or vCCL2, or 2 U/ml of apyrase was analyzed as in A. Bars represent mean ± SD number of migrated cells measured in triplicate from one experiment representative of 3 independent experiments. p-Values are from a 2-way ANOVA test with Tukey correction for multiple comparisons. The underlying numerical values for the panels displaying summary numerical data can be found in S1 Data. (PDF) [file pbio.3001259.s010.pdf]

Uncropped blots for Figure 2

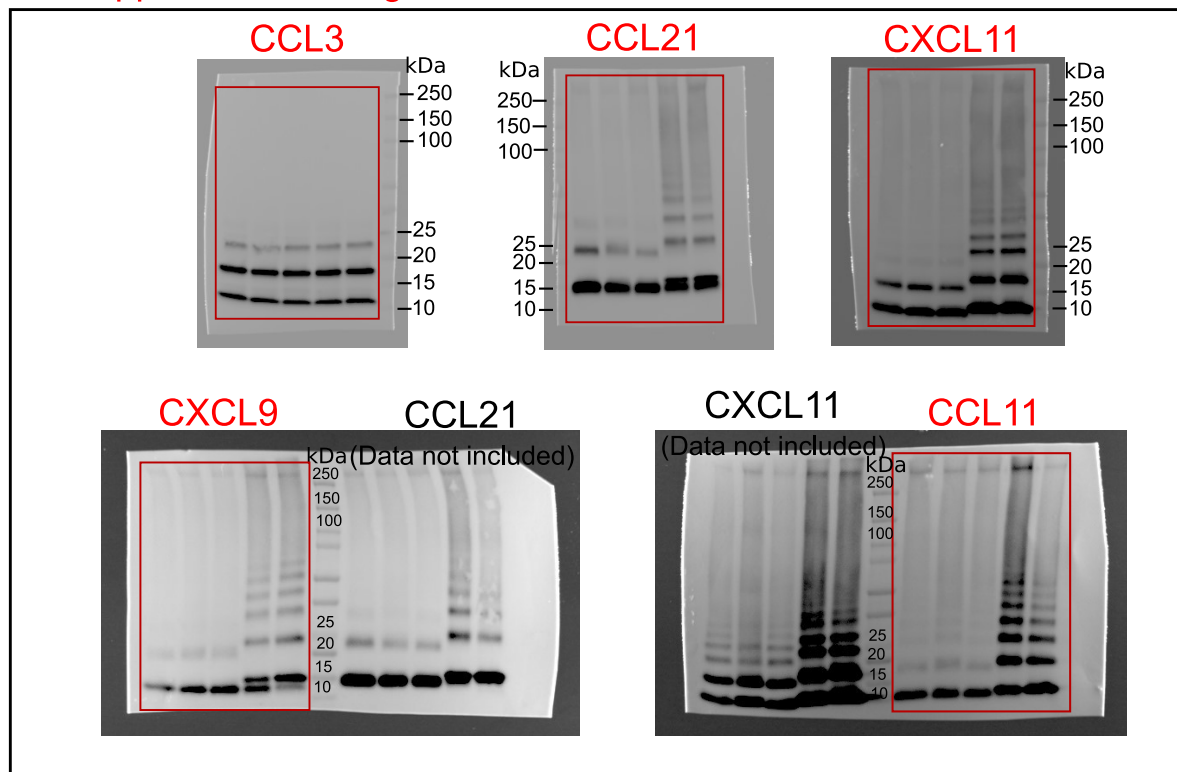

Uncropped blots for Figure 5D

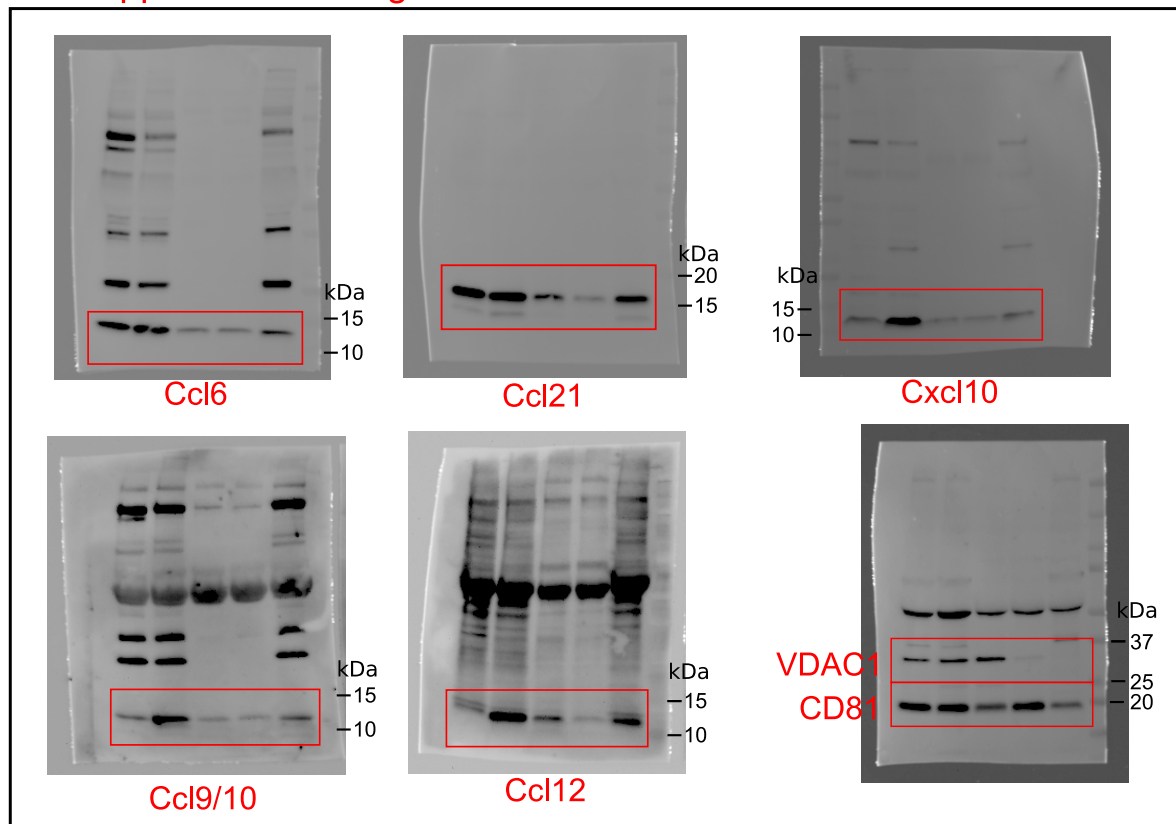

Uncropped blots for Figure 5A

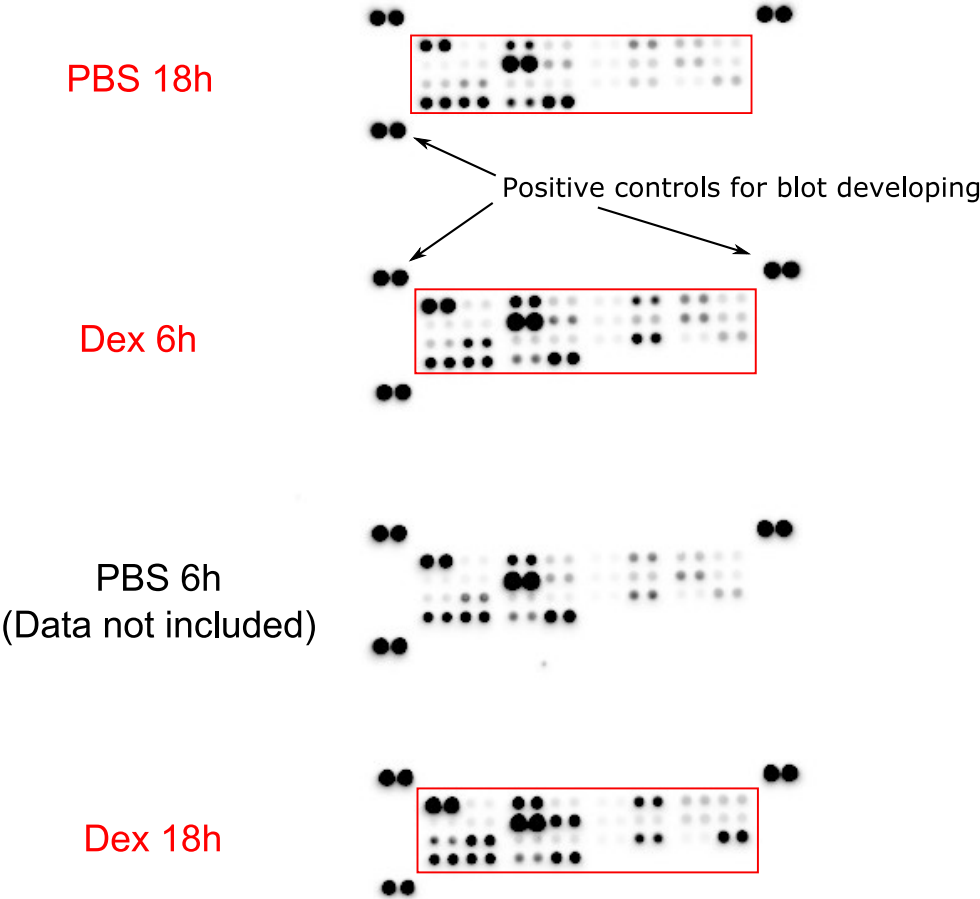

Supplement: S1 Raw Images for Gels and Blots — (PDF) [file pbio.3001259.s014.pdf]
