# Supplementary material for: Chemokines act as phosphatidylserine-bound “find-me” signals in apoptotic cell clearance
Source: PLoS Biol. 2021 May 26;19(5):e3001259. doi: 10.1371/journal.pbio.3001259 (PMC8213124; doi:10.1371/journal.pbio.3001259)
Supplement: S1 Table — Binding fold change relative to the binding of the known PS-binding chemokine CXCL16 is indicated in the third column. Binding responses <0.1 nm are highlighted in orange. Fold change values >2.0 and <0.5 are highlighted in blue or purple, respectively. BLI, biolayer interferometry; PS, phosphatidylserine. (PDF) [file pbio.3001259.s011.pdf]

	Binding at 495 s (nm)	Fold change
**CXCL16**	0.428	1.000
**CCL2**	0.407	0.951
**CCL3**	0.041	0.096
**CCL11**	1.346	3.145
**CCL13**	1.554	3.631
**CCL17**	0.278	0.650
**CCL19**	1.158	2.706
**CCL20**	0.660	1.542
**CCL21**	0.312	0.729
**CCL22**	0.247	0.577
**CCL23**	0.061	0.143
**CCL24**	0.146	0.341
**CXCL1**	0.194	0.453
**CXCL2**	1.559	3.643
**CXCL3**	0.348	0.813
**CXCL4**	2.396	5.598
**CXCL5**	0.140	0.327
**CXCL6**	1.867	4.362
**CXCL8**	0.055	0.129
**CXCL9**	1.142	2.668
**CXCL11**	2.912	6.804
