# Supplementary material for: Chemokines act as phosphatidylserine-bound “find-me” signals in apoptotic cell clearance
Source: PLoS Biol. 2021 May 26;19(5):e3001259. doi: 10.1371/journal.pbio.3001259 (PMC8213124; doi:10.1371/journal.pbio.3001259)
Supplement: S1 File — (PDF) [file pbio.3001259.s012.pdf]

Supporting materials and methods

Expression and purification of recombinant chemokines

Synthetic codon-optimized genes encoding CCL21, CXCL9 and CCL3 were obtained from Life Technologies. A twin-strep tag (TST, seq: SAWSHPQFEKGGGSGGGSGGSAWSHPQFEK) was added at the immediate C-terminus of each chemokine for purification and detection purposes (IBA Lifesciences, Göttingen, Germany). Genes were subcloned into a pcDNA3.1 plasmid for expression of the proteins under a CMV promotor. For the expression of recombinant protein, 150 x10^6 Expi293F cells were transfected with 60 µg of plasmid using the Expifectamine 293 Transfection Kit (Life Technologies) following the manufacturer's instructions. 4-7 days after transfection, protein secreted into the supernatant was purified by affinity chromatography using Strep-TactinXT gravity columns (IBA Lifesciences) following the manufacturer's guidelines. Briefly, cell-free supernatant was buffered with 1X Buffer W (100 mM Tris pH 8.0, 150 mM NaCl, 1 mM EDTA) and biotin in the culture media was blocked with 18.1 ml/L of BioLock Solution (IBA LifeSciences). Subsequently, the supernatant was further clarified by centrifugation (4,000 rpm, 15 min) and applied at a 1 ml/min rate into a Strep-TactinXT gravity column. The column was washed with 5 ml Buffer W and bound protein was eluted with 2 ml Buffer BXT (100 mM Tris pH 8.0, 150 mM NaCl, 1 mM EDTA, 50 mM biotin). Protein-containing elution fractions were pooled together, and concentrated and dialyzed with PBS using 3 kDa Amicon Ultra 0.5 ml Centrifugal Filters (Millipore, Bedford, MA). Protein concentration was determined by SDS-PAGE and densitometry, and proteins were stored at -80° C.

Pull-down of chemokine-liposome complexes

To investigate the relative contribution of free chemokines versus chemokines complexed with liposomes to cell migration, we tested the chemotactic activity remaining in solution after depletion of chemokine-liposome complexes by pull-down. For this, 1 nM CCL3 or CCL20 was

incubated with buffer or a 10^4-fold molar excess of DOPC or DOPS liposomes in 0.4 ml of chemotaxis buffer (DMEM-Glutamax supplemented with 0.1% BSA and 10 mM HEPES) for 30 min at room temperature. Then, a total of 30 µl of Strep-TactinXT-coupled agarose beads (IBA LifeSciences), previously washed 5 times with chemotaxis buffer, was added to each sample and incubated with rotation for 30 min at room temperature. Beads were pelleted by centrifugation (12,000 rpm, 1 min) and the supernatants were collected and tested by ELISA and chemotaxis assays as explained before.

Analysis of the expression of chemokine receptors in apoptotic cells and MM1 monocytes

The expression of chemokine receptors of interest in MM1 cells or apoptotic CHO-745 cells and mouse thymocytes was analyzed by FACS. Apoptosis of CHO-745 and mouse thymocytes was induced as described before. After blocking cell surface Fc receptors with TruStain FcX (Biolegend), cells (5-3 x 10^5) were stained for 20 min on ice with one of the following PE conjugated antibodies (all from Biolegend) in PBS-staining buffer (1X PBS supplemented with 1% BSA and 1% FBS): anti-CXCR4 (clone QA18A64), anti-CCR2 (clone K036C2), anti-CCR7 (clone G043H7), anti-Ccr2 (clone SA203G11), anti-Ccr3 (clone J073E5), anti-Ccr6 (clone 29-2L17), anti-Ccr7 (clone 4B12), anti-Cxcr3 (clone CXCR3-173), or mouse IgG1, mouse IgG2a, rat IgG2a, rat IgG2b, and armenian hamster IgG isotype controls. CHO-745 and mouse thymocytes were subsequently washed with Annexin V (AnV) binding buffer (Biolegend) and stained with AnV-APC (Biolegend). At least 30,000 events were acquired in a LSR Fortessa cytometer and analyzed using FlowJo (both from BD Bioscience).
