# Supplementary material for: Chemokines act as phosphatidylserine-bound “find-me” signals in apoptotic cell clearance
Source: PLoS Biol. 2021 May 26;19(5):e3001259. doi: 10.1371/journal.pbio.3001259 (PMC8213124; doi:10.1371/journal.pbio.3001259)
Supplement: Attachment — Submitted filename: MurphyReview_PS_Binding_CKs.pdf [file pbio.3001259.s015.pdf]

The manuscript "Chemokines as phosphatidylserine-bound 'find-me' signals in apoptotic cell clearance" by Pontejo and Murphy reports an extremely interesting finding regarding the ability of chemokines to bind to and be presented by phospholipids, specifically phosphatidylserine (PS) to chemokine receptors. It adds a twist to the conventional notion regarding the presentation of chemokines by glycosaminoglycans. The interactions are shown to be quite specific including the fact that some chemokines that normally bind with high affinity to glycosaminoglycans (e.g. CCL5) do not bind with high affinity to PS. The authors also show that glycosaminoglycans are significantly depleted on the surface of apoptotic cells or blebs. Another interesting discovery is that in contrast to glycosaminoglycans, PS liposomes do not inhibit the ability of chemokines to recruit cells, suggesting that the molecular determinants for binding PS-chemokine and glycosaminoglycan-chemokine interactions are different. The study is quite comprehensive in exploring many details of this interaction and convincingly demonstrate that the phenomenon is real. Together, the findings lead to a conceptually logical mechanism, whereby cells undergoing apoptosis, expose PS lipids (normally on the inner leaflet of the cell bilayer) which serve to localize chemokines and cause cell migration to clear apoptotic debris. As noted in the discussion, the implications of these finding are potentially quite broad since tumor cells and infected cells also expose PS, and extracellular vesicles which are widely used for intercellular communication in cancer and infectious disease, may also be vehicles for chemokine presentation. For these reasons I highly recommend publication.

Overall, I found the manuscript to be a well written paper, although a few minor changes are suggested.

1. The initial data presented with chemokines binding to lipid arrays and to liposomes using antibody detection versus bilayer interferometry is slightly confusing because the results are inconsistent. Ultimately the authors suggest differences are due to issues with antibody detection of chemokine bound to liposomes in the first two methods vs BLI, but it would be helpful to somehow present the data in a cleaner fashion. I found myself rereading this section several times because of the inconsistent data and then finally got to line 172 which revealed a likely explanation for the inconsistency.

2. It would be helpful if the authors discussed more of the relationship between the PS binding chemokines and the cells they recruit in the context of the apoptotic mechanism. CCL12, is briefly mentioned but what about other chemokines that might be expected to recruit monocytes and macrophages? I do realize that a deeper understanding will require further study beyond the scope of this manuscript (as stated) but nevertheless some additional insights would round out the mechanism.
