# Supplementary material for: Chemokines act as phosphatidylserine-bound “find-me” signals in apoptotic cell clearance
Source: PLoS Biol. 2021 May 26;19(5):e3001259. doi: 10.1371/journal.pbio.3001259 (PMC8213124; doi:10.1371/journal.pbio.3001259)
Supplement: Attachment — Submitted filename: Point by point response.pdf [file pbio.3001259.s016.pdf]

RE: PBIOLOGY-D-20-01298R1
"Chemokines as phosphatidylserine-bound 'find-me' signals in apoptotic cell clearance"

ACADEMIC EDITOR
Our Academic Editor raises a related point that PS is exposed not only by apoptotic cells, which release anti-inflammatory signals, but also by cells dead by necroptosis and other forms of regulated necrosis, which are inflammatory forms of cell death. It is common that, in vitro, cells that start dying by apoptosis then undergo secondary necrosis because they cannot be removed as in the tissue. You will need to address this concern in your revision.

Response: We completely agree. As was also pointed out by the reviewers, our findings have broad multidisciplinary implications (different forms of cell death, cancer and viral infections). As a result, our long-term future goals include investigating each biological context where PS and other chemokine-binding anionic phospholipids are exposed to define the scope and specificity of chemokine binding that might occur and the functional significance. This will include additional forms of cell death beyond the two that we have tested experimentally in the paper, including necroptosis. Moreover, in contexts where necrotic cells cannot be removed it will be interesting to test whether factors that modulate chemokine binding to PS might contribute to this resistance. A full investigation of each of these additional contexts is clearly beyond the scope of the present paper, however. Nevertheless, we have broadened the Discussion to highlight this important point regarding the potential expansive impact of the study.

REVIEWER #1

Point 1: *Materials and Methods: How each chemokine was prepared? Are they native proteins or recombinant proteins? If they are recombinant, are they produced in mammalian cells or E. coli? If they are commercially available non-glycosylated form (Line 283), is it prepared in E. coli. Chemokines carry many cysteine residues. How the authors know the molecules are fully active. Some chemokines are membrane-bound form. Only the extracellular region was used? The authors use antibodies to recognize chemokines. Please describe sources of each antibody.*

Response: We have added a new section to the Materials and Methods entitled "Reagents" (Line 594) where, as requested, we clearly specify the source and nature of our chemokines and antibodies, and that the protein CXCL16 corresponded only to the extracellular domain of human CXCL16. Most of the proteins included in this study were commercial recombinant chemokines. The vendor performs quality validation of all chemokines by chemotaxis assays and provides the ED_{50} for each chemokine. In addition, proof that these chemokines were in fact active can be found in our own results in the panel "agonist" of Fig 5E.

Point 2: *Fig. 1A: The authors use the respective antibodies to detect the chemokines in the array and ELISA assay, which make the comparison between chemokines very difficult. The authors have the system to prepare the strep tagged chemokines (Fig. S4). If the authors want to compare the ability of each chemokine to bind PS, all chemokines should be prepared in the same way and should be detected by the same detection system.*

Response: We agree, which is why we expanded our investigation of chemokine binding specificity to include biolayer interferometry (BLI, Fig 1E), a technique that permits direct analysis and comparison of the binding of many different chemokines in the exact same experimental conditions without the need for exogenous tags on the chemokine and without using detection antibodies. Nevertheless, we included the pure anionic phospholipid array results, because this is the screen that launched our investigation, as well as the ELISA results, since this is how we investigated the effect on binding of a membrane bilayer of different compositions. We have now included new statements to clearly acknowledge the technical limitations inherent in each assay (Lines 140, 148, 164). Repeating the analysis with tagged chemokines would add another variable that we feel is unnecessary given the direct BLI results, and in any case would involve the cloning, expression and purification of 21 different chemokines, which would take many months to complete.

Point 3: *Fig. 1A, 1B/1D, and 1E/1F. Some chemokines bind to PS in one assay (array), but not others (ELISA or BLI). While other chemokines do not bind PS in the array, but binds PS in the other assay. It is very confusing, and indicates that something wrong in the assay or the experimental system.*

Response: As mentioned in the Response to Point 2, the three binding assays are complementary, each providing a different perspective of chemokine binding to phospholipids (phospholipid binding profile by lipid array; binding to PS in liposomes by ELISA and BLI; PS specificity by ELISA; comparison of PS-binding activity of different chemokines by BLI; PS-binding affinity by BLI). Each has its own strengths and its own set of limitations owing to different experimental conditions (different buffers, different washing procedures, different disposition of the lipids, different immobilization surfaces, antibody-based vs antibody-independent detection…). Therefore, it is not surprising that there might be differences in sensitivity. For example, a chemokine with a fast off rate might be missed in the ELISA assay, which involves multiple washes, but not in the BLI assay, which is designed to measure both on rates and off rates. Also, as we show in S2 Fig, some anti-chemokine antibodies may not detect their target chemokine in complex with the lipid, which explains why the interaction is detectable by BLI (antibody-independent technique) but not by ELISA (antibody-dependent technique) (First paragraph Page 8). Despite this, we only found a few discrepancies, which are described in detail in the revised manuscript with discussion of possible explanations drawn from the technical differences among the assays such as the examples mentioned here. All together, these assays provide strong evidence and complete biochemical characterization of chemokine-phospholipid interactions. We thank the reviewer for raising this concern, which we have addressed by rearranging the panels of Fig 1 and by highlighting and discussing the discrepancies in greater detail in this section of the Results (Pages 7 and 8).

Point 4: *Fig. 1E and 1F: I am not sure whether this BLI analysis is giving the correct data or not. Why the response values do not go down to the bottom in the washing step? In some cases (CCL-21, CXCL4 and CXCL9), they even increase. Why? I do not think that the fitting to Langmuir model gives the correct kinetic values under these conditions?*

Response: The binding response detected by BLI, or other sensor-based biophysical techniques like surface plasmon resonance (SPR), does not always go back down to baseline. When an interaction shows a dissociation that quickly returns to baseline, as the one described by the Reviewer, it is indicative of a very unstable complex. In contrast, high affinity interactions like antibody-antigen, or streptavidin-biotin display a dissociation phase where the binding

response goes down very slowly if at all (there are hundreds of examples of this in the literature). The fact that some of our chemokines dissociate from DOPS liposomes very slowly simply indicates that chemokines form very stable complexes with PS, which supports that this interaction may in fact occur in vivo. The opposite observation, a chemokine binding to PS but then quickly returning to baseline, would have raised concerns about the biological significance of such interaction. We agree with the Reviewer that in the binding assay of Fig 1E some chemokines display a dissociation phase where the binding response appears to increase slightly. This is a common artifact of BLI and SPR caused in part by mass transfer effects, which can be attenuated by reducing the amount of immobilized ligand on the sensor, as we did for our kinetic analysis in Fig 1F. However, the presence of this artifact in the dissociation phase of the sensorgram does not invalidate the conclusions of our binding assay in Fig 1E about the presence or absence of chemokine binding to PS. We agree with the Reviewer that in Fig 1F the Langmuir model does not show a flawless fitting to the binding curves. In these experiments, we analyzed the binding of chemokines to a heterogeneous ligand, liposomes containing DOPC and DOPS, but since we removed the effect of DOPC by subtracting the binding to pure DOPC liposomes, and in the absence of knowledge about the stoichiometry of the chemokine-PS interaction, we concluded that a general 1:1 Langmuir model was the most appropriate option. Other fitting models like local adjustment or 2:1 provided more esthetic fittings but utterly unrealistic $KD<10^{-12}$ M, very close if not beyond the detection limit of the instrument.

Point 5:*Fig. S1: How MFG-E8 is prepared? Please show the Kd for the interaction of MFG-E8 with PS.*

Response: As indicated now in the new “Reagents” section of Materials and Methods (Line 594), recombinant human MFG-E8/lactadherin was purchased from R&D Systems. The affinity of the binding of MFG-E8 to PS has been previously published by others. As requested, this information and the appropriate reference have been added to the revised version of the manuscript (Line 181).

Point 6: *Fig. 2: PS-induced oligomerization is interesting. Please describe the experimental procedure in more detail. Phospholipids are usually insoluble in water, and are dissolved in CHCl3 and methanol. How phospholipids were mixed with chemokines in this experiment? The Mr of phospholipids are about 1,000. When the authors say that chemokine and phospholipids are mixed at 1:8, the 50 ng of chemokines were mixed with 50 ng of phospholipids? What is the mechanism of the oligomerization? Or, how phospholipids cause the oligomerization of chemokines?*

Response: As indicated in Material and Methods, in these experiments we mixed 50 ng of chemokine with an 8-fold molar excess of phospholipid, approximately 30-60 ng of phospholipid depending on the MW of the different chemokines and phospholipids. Since our phospholipid stocks were stored in chloroform at 10 mg/ml, we needed to use only 0.003-0.006 μl of phospholipid, which we achieved by simply diluting 1 μl of phospholipid into 1 ml of 20 mM HEPES and then transferring 3-6 μl of this dilution into the chemokine mix. The details of this initial dilution of our phospholipid stocks are now shown in the revised version of Materials and Methods (Line 682). 1 μl of chloroform is soluble in 1 ml of an aqueous buffer such as 20 mM HEPES (chloroform water solubility is 0.008 g/ml, 1 μl corresponds to 0.00149 g of chloroform) and when transferred into polar solvents, phospholipids spontaneously form micelles, which are stable and soluble in water and other polar solvents. We agree with the reviewer that it would be

interesting to understand the mechanism of chemokine oligomerization on phospholipids (See also our response to Reviewer's Point 7). This question will be addressed in our future research since the full and detailed analysis that this requires is beyond the scope of the present paper.

Point 7: *Fig. 3: With the CXCL16 molecules that carry mutation in the conserved receptor-binding domain (chemokine-domain), Shimaoka, T. et al., (reference 15) showed that chemokines (at lease CXCL16) binds to oxidized LDL via the receptor binding domain. Here, the authors claim that the PS-engaged chemokines can have the chemo-attractive activity. To strength this conclusion, a mutational analysis is necessary. Or, please show the mutants that cannot bind PS still have the chemo attractive activity.*

Response: In the chemokine-PS complex, the PS binding sites of the chemokine are occupied and masked by PS molecules. If PS-bound chemokine can activate its cognate cellular receptor, one can conclude that PS-binding does not affect chemokine activity and that the chemokine PS-binding sites are not required for the chemokine interaction with the receptor. We provide several lines of evidence to support this conclusion: i) Increasing doses of DOPS liposomes, up to a very high 10^5 molar excess, do not reduce chemotactic activity (Fig 3A left column); ii) A high 10^4 molar excess of DOPS liposomes does not affect the chemokine chemotactic dose response (Fig 3B); iii) PS-binding chemokines interact with DOPS liposomes in these experimental conditions (Fig 3A right column); iv) removal of chemokine-liposome complexes by pull-down reduces chemotaxis induced by PS-binding chemokines (S4 Fig); v) PS-binding chemokines bound to apoptotic bodies and microvesicles extracted from mouse apoptotic thymus activate their cognate cellular receptors (Fig 5 and S9 Fig). All this together demonstrates that PS-binding does not affect chemotaxis. In contrast, we show that soluble heparin inhibits chemotaxis induced by CCL20 and CCL21 (Fig 3B). Increasing structural evidence has shown that heparin binding sites of chemokines overlap with their receptor binding site, which validates our postulate above that if a chemokine ligand such as PS permits chemokine normal activation of a cellular receptor then, any physical overlap in the binding sites for that ligand and the receptor on the chemokine, by definition, is not functionally significant. It has taken years of collaborative research and many publications to understand how chemokines interact and oligomerize with glycosaminoglycans (GAGs) and to realize that these activities often involve a different molecular mechanism in each chemokine. As we mention in the Discussion, this is likely to be the case for PS-binding as well, and multiple studies of the PS-binding activity of different individual chemokines will be required to understand how chemokines interact and oligomerize (to the Reviewer's Point 6) with phospholipids. The detailed analyses that will be required are beyond the scope of the present paper.

Point 8: *Line 274, Fig. 4A: PI+ AnV+ cells are NOT apoptotic cells. PI+ means that cells underwent necrosis with ruptured plasma membranes. Please see Crowley et al. (CSHL Protoc, doi: 10.1101/pdb.prot087288).*

Response: While, as indicated by the Reviewer, some may refer to PI+ cells as necrotic cells regardless of their AnV staining, many others in the field designate PI- AnV+ cells as early apoptotic cells, PI+ AnV+ cells as late apoptotic cells and PI+ AnV- cells as necrotic cells. Of note, increasing evidence indicates that a simple PI/AnV staining is not enough to determine if a cell dies by apoptosis, pyroptosis, necroptosis, necrosis of other molecular mechanism of cell death. To avoid implying that in our experiments UV-irradiated CHO-745 cells died by any particular mechanism, we now clearly refer to these PI+ AnV+ CHO-745 cells as "late apoptotic

cells" in the revised manuscript (Page 12), in contrast to the PI- AnV+ early apoptotic thymocytes of Fig 4D-F.

Point 9: *Reference 28 should be replaced by Hanayama et al. (Nature 417, 182, 2002).*

Response: Reference has been replaced as requested.

REVIEWER #2:
This is an interesting and conceptually novel observation, which extends our knowledge on the chemokine system. All experiments are conclusive and build a logical line to support the hypothesis. I have only minor suggestions to complete the manuscript.

Response: We thank the Reviewer for these comments.

Point 1: *Figure 3: at least for one chemokine a dose response curve (CK) in the presence of different DOPS/DOPC ratios should be given.*

Response: Fig 3B shows that a constant 10^4 molar excess of liposome has no effect on the chemotaxis induced by increasing doses of chemokines (0.1-100 nM). Therefore, we can reasonably conclude that a lower dose of liposome (10^2 or 10^3 molar excess) will not affect chemotaxis either. The effect of a higher 10^5 liposome molar excess on 1 nM of chemokine is already shown in the left column of Fig 3A. 1 nM was carefully chosen as the optimal concentration of chemokine that would allow us to detect any possible inhibition by liposomes. In Fig 3B "buffer" curve, it can be seen that 1 nM is perfectly placed in the chemotaxis dose response curve of every chemokine tested to detect potential inhibitory effects. If 10 nM had been chosen instead, notice that a 10-fold reduction in the CCL20 availability by a putative inhibitor would have resulted in the same levels of chemotaxis (compare # migrated cells at 10 nM vs 1 nM CCL20 in the "buffer" curve). At the higher 100 nM concentration, CCL20 and CCL3 do not induce migration (Fig 3B), plus, at this chemokine concentration, it would be technically very challenging to prepare liposomes to be added at a 10^5 molar excess (liposomes would need to be added at a final 10 mM concentration). For these reasons, we consider that repeating the experiments of Fig 3B with a liposome molar excess other than 10^4 would not provide any additional information to support our conclusion that PS containing liposomes do not interfere with chemokine activity.

Point 2: *Figure 4: The effect of UV irradiation (panel A, right column AnV/PI) should be quantified. The quadrants should indicate % of cells. Panel B: should indicate the absolute cell numbers. From the current presentation it is difficult to appreciate the increase in chemokine binding after UV irradiation.*

Response: Dot-plots in the right column of Fig 4A are overlays of the PI/AnV staining of the FSC^{lo} and FSC^{hi} cells gated in the left dot-plots in orange and blue, respectively. These overlays show that almost all FSC^{lo} cells were PI+ AnV+ and almost all FSC^{hi} cells were PI- AnV-, and therefore, the FSC profile could be used to distinguish live (FSC^{hi}) and dying cells (FSC^{lo}) without the need of any other apoptosis marker in the subsequent chemokine binding experiments. The effect of UV irradiation on CHO-745 cells is shown and quantified in the left

column of Fig 4A: increase of FSC^{lo} cells (dying cells) from 10.3% to 34.7%, and increase of SSC^{lo} events (apoptotic bodies) from 0.6% to 19.8%. We do not claim that there is an increase in chemokine binding after UV irradiation. On the contrary, as shown in Fig 4B, the binding of CCL11, CXCL11, and AnV, to FSC^{lo} cells from mock-treated samples (Mock-FSC^{lo}) was comparable to their binding to FSC^{lo} cells from UV irradiated cells (UV-FSC^{lo}). The goal of these experiments was to compare chemokine binding to FSC^{lo} (dying cells, orange histograms) and FSC^{hi} (live cells, blue histograms), and in this regard, our results show a clear binding (> 2 log difference) of CCL11 and CXCL11 but not of CCL3, consistent with their PS binding activity. However, we agree that the way we described these results in the text may be confusing. In the revised version of the manuscript we have rewritten this part of the Results to describe these results and our conclusions more clearly (Page 12).

Point 3: *Panel E needs proper statistical analysis.*

Response: Statistical analysis has been applied to Fig 4E as requested.

Point 4: *Line 335 4E should read 4F*

Response: This was corrected as requested (now in Line 350).

REVIEWER #3:
This is a very interesting, and well-written, manuscript which provides important new insights into aspects of in vivo chemokine cellular presentation and function. Overall I am enthusiastic about this paper but I have a number of concerns many of which are more philosophical in nature and can possibly be dealt with by an enhanced discussion.

Response: We thank the Reviewer for these comments. As requested, the Discussion has been enhanced with additional comments addressing the points made by the Reviewer and with a graphic representation of the proposed model in a new Fig 6.

Point 1: *I am not persuaded that the data clearly indicate a role in apoptotic cell clearance as stated in the title. Certainly this is an attractive model but many of the chemokines that are highlighted as having strong PS binding (e.g. CCL20, CCL21 CXCL11 etc) would not be expected to attract phagocytes and therefore their role as PS bound 'eat me' signals seems unlikely. I wonder whether there is a broader biology here that might be worth considering.*

Response: The Reviewer correctly states that chemokines that do not act on phagocytes would not be expected to have an impact on apoptotic cell clearance. However, as we discuss, PS-exposure is not unique to apoptotic cells. Tumor and virally infected cells as well as microvesicles derived from these cells are also known to expose PS in the plasma membrane. Therefore, PS-mediated binding to microvesicles may play an important role in the regulation of chemokines that attract other cell types in cancer and infectious diseases. These ideas are now more clearly presented in the revised Discussion (Pages 20 and 21). See response to Point 4 below for our response to the Reviewer's concerns about the title.

Point 2: *Another point is that, in terms of attracting phagocytes to the 'eat me signals' this would imply a gradient of sorts from the apoptotic cell to the phagocyte. This then raises the question of where the PS-bound chemokines are coming from. If they are coming from another source then it is unlikely that their binding by apoptotic cell surface, and microvesicle, PS would allow a gradient to form that would attract phagocytes to the dying cell. In contrast, if the chemokines are actually produced by the dying cell then this might be a very attractive model and allow the apoptotic cell to be the chemokine 'source' and not a 'sink'. As apoptotic cells are likely to be producing inflammatory chemokines, including attractants for monocytes and macrophages (see figure 5A), this may give much more specificity to this axis in terms of attracting phagocytes (although I accept that many of these chemokines are not good PS binders). I suspect that it would be very difficult to formally prove this idea and so I am not asking for experiments to be done in this regard but this at least needs to be discussed.*

Response: We have extended the Discussion to address the Reviewer's very important point about the origin of the chemokines (Line 515). We also agree that PS binding to apoptotic cells could serve a chemokine scavenging function has to be considered as future research into the biology of this goes forward (See also response to Point 3). We have included a new Fig 6 with a graphic representation of the proposed model described in the Discussion.

Point 3: *In terms of broader biology is also possible that the exposure of PS on the surface of apoptotic cells, and on apoptotic microvesicles, could act in a similar way to circulating ACKR1 i.e. as a chemokine 'sponge' mopping up chemokines and contributing to resolution of inflammatory and immune responses. This may help further explain the strong binding of non-phagocyte-attracting chemokines and certainly is worth discussing in the text.*

Response: Thanks for raising this important point, which we had not previously emphasized but have now included in the Discussion (Line 522 and Fig. 6).

Point 4: *Overall, therefore, this is an interesting paper but I am not persuaded that the results clearly indicate a role in apoptotic cell clearance as suggested by the title.*

Response: Find-me signals are vaguely defined as apoptotic cell-derived factors that exert chemotactic activity on macrophages or other professional phagocytes. Since their study in vivo has proved to be very difficult, the characterization of find-me signals has been greatly restricted to in vitro systems. Several studies have reported that apoptotic bodies and blebs attract macrophages, and therefore, the definition of find-me signal is applicable and has been applied to these extracellular vesicles. However, the molecular mechanism of this apoptotic body-mediated recruitment of macrophages has remained poorly understood. Here we demonstrate that Ccl12, ligand for Ccr2, a chemokine receptor expressed by macrophages, is induced in mouse apoptotic thymus, binds PS and associates with apoptotic vesicles to activate Ccr2, unlike chemokines unable to interact with PS (See Ccr1 ligands in Fig 5). In the revised manuscript, we include new experiments that demonstrate that removal of apoptotic blebs by filtration or inhibition of Ccl12 with a neutralizing antibody obliterate the activation of Ccr2 by apoptotic body-containing fractions isolated from apoptotic thymus (new S9 Fig). Therefore, apoptotic bodies per se do not activate Ccr2-expressing cells, they need to be in complex with Ccl12. We did not observe induction of other Ccr2 ligands like Ccl2 in mouse apoptotic thymus, but other PS-binding macrophage-attracting chemokines could be expressed in different tissues or cell types during apoptosis. Furthermore, we demonstrate that human CCL2 or CCL13 (CCR2 ligands in human) interact with PS. Our study provides proof of principle that these other

chemokines could associate with apoptotic blebs via PS to recruit CCR2+ macrophages. In addition, as now discussed in the revised manuscript, future efforts could identify other chemokine receptors induced in macrophages and other phagocytic cells during apoptotic cell clearance, expanding the array of chemokine-apoptotic body complexes that may function as find-me signals. We hope the additional experiments and the enhanced Discussion in the revised manuscript help to better convey the rationale of the paper's title.

Point 5: *Statistical analysis should be improved throughout. The analysis of figures 4D, 5F and supplementary figure 3A in particular requires proper statistical testing and indication of significance levels. These figures are fundamental to the conclusions being drawn and therefore this analysis is essential.*

Response: Statistical analyses have been applied to all pertinent figures as requested.

REVIEWER #4:

For these reasons I highly recommend publication.
Overall, I found the manuscript to be a well written paper, although a few minor changes are suggested.

Response: We thank the Reviewer for these comments.

Point 1: *The initial data presented with chemokines binding to lipid arrays and to liposomes using antibody detection versus bilayer interferometry is slightly confusing because the results are inconsistent. Ultimately the authors suggest differences are due to issues with antibody detection of chemokine bound to liposomes in the first two methods vs BLI, but it would be helpful to somehow present the data in a cleaner fashion. I found myself rereading this section several times because of the inconsistent data and then finally got to line 172 which revealed a likely explanation for the inconsistency.*

Response: To improve the clarity of this section of the Results flagged by the Reviewer, we have rearranged the panels of Fig 1 and edited the description of these results in the text of the revised manuscript (Pages 7 and 8).

Point 2: *It would be helpful if the authors discussed more of the relationship between the PS binding chemokines and the cells they recruit in the context of the apoptotic mechanism. CCL12, is briefly mentioned but what about other chemokines that might be expected to recruit monocytes and macrophages? I do realize that a deeper understanding will require further study beyond the scope of this manuscript (as stated) but nevertheless some additional insights would round out the mechanism.*

Response: The Discussion has been extended to include other chemokines that recruit macrophages as well as to clearly present the potential role of PS-binding in the regulation of other chemokines with non-phagocyte target cells (Pages 20 and 21). In addition, a new Fig 6 with a graphic representation of the proposed model has been included to support the Discussion.
