# Supplementary material for: Chemokines act as phosphatidylserine-bound “find-me” signals in apoptotic cell clearance
Source: PLoS Biol. 2021 May 26;19(5):e3001259. doi: 10.1371/journal.pbio.3001259 (PMC8213124; doi:10.1371/journal.pbio.3001259)
Supplement: Attachment — Submitted filename: Response to reviewers revision 2.pdf [file pbio.3001259.s017.pdf]

RE: PBIOLOGY-D-20-01298R2
"Chemokines as phosphatidylserine-bound 'find-me' signals in apoptotic cell clearance"

REVIEWER 2
The authors have answered all my concerns. No alterations requested. Given that migrating leukocytes internalize chemokines during chemotaxis, it would be interesting in the future to reveal whether chemokines dissociate from liposomes/apoptotic blebs or are internalized with the lipid vesicles.

Response: We thank the Reviewer for the positive evaluation and suggestion.

REVIEWER 3
All comments have been satisfactorily addressed. I am still a little uncomfortable with the title but the biology is of sufficient interest that I will not request any further changes.

Response: We thank the reviewer. We think the new experiments presented herein (Fig 6) support the title.

REVIEWER 4
The authors have done a nice job of responding to reviewer concerns with appropriate changes to the manuscript. The inclusion of the model (Fig 6) is a nice addition. I recommend publication.

Response: We thank the Reviewer.

REVIEWER 5
While the binding of chemokines (other than CXCL16) to PS interesting, the physiological relevance of these findings is questionable. Indeed, and in contrast with the title of the manuscript, the authors do not provide any results demonstrating a role in apoptotic cell clearance. The results therefore appear very preliminary for the claims made by the authors.

Response: This concern has been addressed experimentally as summarized below and as shown in a new Fig 6.

Point 1: *Figure 2. It is not clear why the authors used a cross linker, as it might artefactually aggregate the chemokines.*

Response: Crosslinking has been traditionally used in the chemokine field to study chemokine oligomerization. However, when initially planning the experiments presented in Fig 2, we contemplated this very same concern raised by the Reviewer. This is why we included numerous controls in these experiments: phospholipid-binding (CXCL9, CXCL11, CCL11 and CCL21) and non-binding (CCL3) chemokines, absence (-) and presence of phospholipids, zwitterionic (PE and PC) and anionic phospholipids (PS and CL). Although the crosslinker was added to all the conditions tested, a clear laddering effect, as a result of the formation of chemokine oligomers of increasing order, only appeared when a phospholipid-binding chemokine (CXCL9, CXCL11, CCL11 and CCL21) was mixed with an anionic phospholipid (PS or CL). In contrast, these same anionic phospholipids did not alter the oligomeric state of CCL3 nor did zwitterionic phospholipids (PE and PC) modify the oligomerization of phospholipid-

binding chemokines when compared to the chemokine oligomers crosslinked in the absence of phospholipid (lane "-") in each case. For this reason, we are confident that the enhanced oligomerization observed selectively for phospholipid-binding chemokines in the presence of anionic phospholipids is specific and not artefactually provoked by the crosslinker.

Point 2: *Figure 3. What is the effect of PS or PC alone on migration?*

Response: As requested, we have included new experiments showing that there is no cell migration when cells are incubated with PS or PC liposomes alone (panel A in S4 Fig).

Point 3: *Figure 4. A-C, The authors claim binding of the chemokines to GAG-deficient late apoptotic CHO cells, but PI+/AnV+ cells are necrotic and not apoptotic. These CHO cells may – or may not - originate from late apoptotic cells undergoing secondary necrosis. In order to know, shorter kinetics of UV treatment should be performed. The authors also refer to apoptotic blebs, but do not characterize them.*

Response: As requested, we now refer to CHO PI+/AnV+ cells as necrotic cells in the text (see lines 293, 295, 298,…) and in Fig 4. We used UV irradiation simply as a method to induce and enhance PS exposure on CHO cells and generate a cellular system where we could test the chemokine-PS interaction. The detailed and multivariate analysis required to determine the mechanism of death of UV-irradiated CHO cells is out of the scope of this study. We address the Reviewer's concern regarding the apoptotic blebs in our response to Point 5 (see below).

Point 4: *Figure 4B, The absence of increase binding in response to UV treatment certainly questions the notion of preferential binding to PS exposed by dead cells.*

Response: We respectfully disagree with the Reviewer that a binding increase in response to UV treatment is required in order to demonstrate preferential binding to dead cells by PS-binding proteins. UV treatment clearly increased the ratio of necrotic cells but, as we explain now more clearly in the text (lines 300-303), we also found a small but measurable % of AnV+ PI+ necrotic cells in mock-treated samples that in terms of PS-exposure were equivalent to the UV-induced necrotic cells (see Fig 4A). In fact, as shown in the bottom row of Fig 4B ("AnV-bt"), there was no difference in the binding of annexin V to necrotic cells from mock-treated or UV-irradiated samples (compare AnV orange histogram in the "Mock" column with the orange histogram in the "UV" column). If the archetypical PS-binding protein displayed no increase in its binding to UV-induced necrotic cells compared to that detected on necrotic cells spontaneously generated in mock samples, it seems reasonable to expect no difference in the binding of any other PS-binding protein either. Nevertheless, the pertinent analysis to conclude that a PS-binding protein displays preferential binding for dead cells is the comparison of the protein binding on PS-exposing dead cells versus on PS-negative live cells. As shown in Fig 4B, while the difference in the binding of CCL3, which does not interact with PS (see Fig 1), to live (cyan histogram) and necrotic cells (orange histogram) was marginal (Fig 4B, top row), annexin V and the PS-binding chemokines, CCL11 and CXCL11, displayed at least a 2-log stronger binding to necrotic cells regardless of treatment. This indicates that PS-binding chemokines and annexin V preferentially bind to PS-exposing necrotic cells. We have rewritten some parts of this section of the Results to improve clarity (Page 12 and 13). In addition, although we found a perfect association between the annexin V staining and FSC profiles of live and necrotic CHO cells (AnV- PI- cells were FSC^{hi} whereas AnV+ PI+ cells were FSC^{lo}, respectively, see Fig 4A) and

previously used the FSC profile to designate live and necrotic cells, in view of this Reviewer's comment, we now think the FSC^{lo}/FSC^{hi} nomenclature may be confusing for the reader. To fix this, we now refer to the different CHO cell populations directly as "live" and "necrotic" cells in the text and Fig 4.

Point 5: *Figure 4C, the authors should do a better characterization of apoptotic blebs. SSC on log scale, size beads, ImageStream is standard analysis to demonstrate vesicular nature of blebs (not just debris on the cytometer): PI low??*

Response: As requested, data have been reanalyzed using log SSC plots to exclude debris from the analysis.

Point 6: *Figure 4E, The results show much stronger binding to live thymocytes than to early apoptotic ones, which does not really fit with the proposed model. The authors try to explain it by the levels of GAG*
expression in live cells, but how does this solve the physiological problem? Where would specificity in the response come from then? Also, not clear what the statistical analysis refer to or means. The legend states "Bars represent mean ± SD of duplicates from one experiment representative of 3 independent experiments"!! Also, in order to demonstrate GAG- and cognate receptor-independent binding of the chemokines to early apoptotic cells, the authors should have performed binding assays with GAG-deficient cells KO for the respective receptor.

Response: As introduced and discussed in the manuscript, it is very well established that cell surface GAGs constitute high affinity binding ligands for most chemokines. Cell surface GAGs are abundant and ubiquitous in all cell lines and primary cells, therefore, we knew beforehand that chemokine binding to cellular GAGs would most certainly interfere with our ability to detect/analyze direct chemokine binding to PS on the surface of apoptotic cells (see line 285). This is why we first performed these experiments with CHO-745 cells, a cell line genetically engineered to lack cell surface GAGs, which, in essence, is the very same control the Reviewer is requesting here, GAG-deficient cells that also lack the cellular receptors for the chemokines of interest (see panel A in S6 Fig).

Unfortunately, UV-irradiation of CHO-745 cells only produced necrotic cells. For this reason, and in order to extend our analysis to primary cells, we decided to test our hypothesis in a better characterized model of apoptosis, dexamethasone-treated mouse thymocytes (Fig 4D-4I). However, unlike CHO-745 cells, thymocytes have GAGs. This is why, although not explicitly requested by the Reviewer, in order to further demonstrate PS-mediated chemokine binding to apoptotic thymocytes, we now include new experiments (Fig 4H and 4I) showing chemokine binding to live and apoptotic thymocytes after treatment with proteinase K, which eliminates cellular GAGs (by cleavage of the proteoglycan protein core that anchors GAGs to the cell surface) but not PS. These new results show that at least 50% of the CXCL11 interaction with apoptotic thymocytes is mediated by PS, and together with the data in Fig 4E, where, as now more clearly explained in the legend, bars represent mean ± SD of the median fluorescence intensity of the binding detected for the indicated chemokines, and in Fig 4F showing that chemokine binding to apoptotic thymocytes was competed by 2 specific PS-binding proteins (Annexin V and MFGE8/lactadherin) without affecting chemokine binding to live cells, prove the main conclusion of this figure, that PS-binding chemokines can exploit membrane-exposed PS to interact with the surface of apoptotic cells.

The fact that even in the presence of live cells abundant in surface GAGs (chemokines were added to a mix of live and apoptotic cells, these populations were not sorted after treatment with dexamethasone, see Fig 4D), like one expects to find in tissues, a significant portion of the chemokine bound to apoptotic thymocytes via PS, supports that the PS-chemokine interaction could also occur in vivo. In addition, this PS-chemokine interaction would be increasingly favored with higher rates of apoptosis because, as we demonstrate for the first time here, apoptotic cells present markedly decreased levels of surface GAGs (see new Fig 4G, and lines 399-406). Furthermore, as supported by increasing structural evidence (frequently discussed in the paper), and as we demonstrate in Fig 3, many GAG-bound chemokines are not able to simultaneously activate their cognate cellular receptors. In contrast, as we show in Fig 3, PS-binding does not interfere with the chemokine activity. Therefore, in the instance of an apoptotic body or apoptotic cells carrying both GAG-bound and PS-bound chemokines on their surface, the PS-bound fraction of the chemokine would be more readily available to interact with cellular receptors and induce cell migration. This is further supported by our results in Fig 5, where we show that extracellular-vesicle enriched fractions (ApoBD and MV) isolated from mouse thymus containing the mouse Ccr1 ligands Ccl5, Ccl6 and Ccl9/10, which do not bind PS (Fig 5B) but can be expected to bind GAGs (Ccl5 is a known strong GAG-binder), do not induce chemokine-like responses in Ccr1-expressing cells (Fig 5E and 5F). In contrast, these extracellular vesicles activated calcium flux and chemotactic signals in cells expressing the cognate receptors for the PS-binding chemokines Ccl12, Ccl21 and Cxcl10 (Fig 5E and 5F). This is now more clearly discussed in the Discussion (lines 678-701, see also lines 460-473) and illustrated in the model of Fig 7.

The manuscript has been edited accordingly to include the description and the discussion of the new results in Fig 4G-4I.

Point 7: *Figure 5. D, markers should be used by WB to differentiate the factions. CCl2 does not seem to be induced, in contrast to the 2x increase reported in A.*

Response: As requested, markers have been included to differentiate ApoBD and MV fractions in Fig 5D. The chemokine highlighted by the Reviewer, Ccl2, was not one of the chemokines we analyzed in Fig 5D. However, we would like to note that the goal of the western blots in Fig 5D was solely to explore whether or not chemokines were present in the ApoBD, MV, and SN fractions. For this purpose, blot developing and image exposing times were optimized to clearly visualize the fainter bands in the ApoBD and MV lanes, which certainly obscured any band intensity difference between the “PBS total” and “DEX total” of chemokines with high basal expression levels such as Ccl6 and Ccl21 (Fig 5D; notice that the high expression levels of these two chemokines in the PBS-inoculated mice are also captured in Fig 5A). Nevertheless, these western blots are not intended for quantitative determinations and no quantitative conclusions are made in the manuscript from these blots. All quantitative conclusions in the manuscript are extracted exclusively from the experiments in Fig 5A, which are more appropriate for this purpose.

Point 8: *Figure 4E-F, similar experiments should have been performed out of the PBS injected control mice.*

Response: We understand the Reviewer is referring to Fig 5E-F instead of Fig 4. The goal of these experiments was to compare the activity of soluble chemokines in the SN fraction with the vesicle-associated chemokine activity in the ApoBD and MV fractions. Since Fig 5A indicated

that the basal expression levels of some of the 6 chemokines of interest could be too low in PBS-inoculated mice to be reliably detected by functional assays, we decided to focus only on the fractions isolated from dexamethasone-treated mice. This is now clearly explained in the text (line 474-476), and as requested, exemplified in a new panel A in S9 Fig.

Point 9: *Figure 4F, L1,2 cells?? These are not a phagocytes – model migration but do not model the complex interaction of a macrophage/DC/phagocyte with an apoptotic target.*

Response: By definition, find-me signals are molecules that attract phagocytes in the context of apoptosis. However, find-me signals do not directly affect phagocytosis per se, which is regulated by “eat-me” signals instead. Once find-me signals bring phagocytes to the vicinity of apoptotic cells, they have fulfilled their most important mission in the process of apoptotic cell clearance and eat-me signals take over to initiate phagocytosis. Since we are claiming that vesicle-bound chemokines are responsible for the find-me signal activity attributed to apoptotic blebs by several independent reports, cell migration was the most important functional response to demonstrate in our study. However, we completely agree with the Reviewer that L1.2 cells are not phagocytes and therefore, we had failed to demonstrate that our findings were relevant for the recruitment of phagocytes, the activity the defines all find-me signals. We have corrected this major flaw of the paper and we now demonstrate, with a set of entirely new experiments in Fig 6, that apoptotic bodies recruit phagocytes in a chemokine-dependent manner.

Point 10: *Figure 6. Not really a summary of the findings but rather a speculative model. The authors represent the chemokine associated to PS in apoptotic blebs recognized by phagocytes as originating from apoptotic cells, but the cellular source of chemokines is not at all demonstrated*

Response: This model is not intended as a summary of the results but rather as a hypothetical model to how all the notions discussed in the Discussion may intersect in the process of phagocyte recruitment in the context of apoptosis. We now make this clear in the legend of the figure. Of note, we added this model after receiving from Reviewer 3 and Reviewer 4 several requests and suggestions of hypothetical elements they considered should be discussed in the paper. In fact, one of those suggestions included the cellular source of the chemokines, which Reviewer 5 highlights in this comment. We already discussed in the previous version of the paper that this remained an open question, but we now clearly state in this revision that the source of vesicle-bound chemokines remains to be identified (line 709). We also agree with the Reviewer that the previous version of this graphical model seemed to indicate that only apoptotic cell-derived chemokines bind to apoptotic blebs and activate phagocyte migration. This has been corrected in the revised model (Fig 7).

Point 11: *Discussion. Line 502: highly atypical chemotactic cues… what about nucelotides? Metabolites? Negates a lot of research on the nature of find me signaling.*

Response: We apologize for this statement. This line has been removed from the text.

Point 12: *Line 500: “these apoptotic blebs only activate cognate GPCRs of PS-binding chemokines*
Of course...” because this is in their in vitro models using transduced GPCRs on B cells .

Response: We think that this statement is clearly supported by our results in Fig 5, where we show that thymic apoptotic bleb fractions (ApoBD and MV), despite containing the Ccr1 ligands, Ccl5, Ccl6 and Ccl9/10 (Fig 5D), which lack PS-binding activity (Fig 5B), are unable to activate Ccr1-expressing cells (Fig 5E and 5F). In contrast, these apoptotic blebs induced strong calcium flux and chemotactic responses in cells expressing Ccr2, Ccr7, and Cxcr3, receptors for the PS-binding chemokines Ccl12, Ccl21, and Cxcl10, respectively. However, as we mentioned above, we agree with the Reviewer that showing that vesicle-bound chemokines were relevant for phagocyte migration was critically missing in the paper. This has been corrected with the new results in Fig 6.

Point 13: *Line 517: live cell-derived chemokines might be expressed at lower levels and might not attract phagocytes. Highly speculative!*

Response: This statement has been removed.

Point 14: *Line 530: the complex of thymus apoptotic blebs with mouse Ccl12 is required to activate mouse Ccr2-expressing cells. 'required' for B cell activation!*

Response: As mentioned above, we now show that vesicle-chemokine complexes are essential for the migration of relevant types of phagocytes (Fig 6).

Point 15: *General comment. The authors have overstated their conclusions based on the data present in the paper. A role for PS-binding chemokines in apoptotic cell clearance is purely speculative based on their data. Critically, the authors should address the capacity for PS-bound chemokines to modulate migration of phagocytes (which will interact with an apoptotic body or cell through the engagement of multiple surface receptors, namely receptors for PS and opsonins), or indeed to affect efferocytosis by phagocytes.*

Response: As mentioned in several previous points, we completely agree with the Reviewer that the absence of evidence that vesicle-bound chemokines are relevant for phagocyte migration was a critical flaw of the previous version of the paper. However, this has been corrected and we now confirm previous observations that apoptotic bodies are strong chemoattractants for monocytes and macrophages, demonstrate for the first time that apoptotic bodies generated in vivo recruit phagocytes, and identify that this find-me activity of apoptotic bodies is mediated by endogenous vesicle-bound chemokines (Fig 6). In addition, we now detail in the Discussion the several lines of evidence provided by our data that support PS-bound chemokine as the most relevant form for vesicle-mediated phagocyte chemotaxis (lines 678-701). The only technically feasible approach we can envision to establish the implications of the PS-binding activity of chemokines in efferocytosis would be the generation of a mouse model expressing a functional chemokine defective in PS-binding (see lines 701-705). An arduous multidisciplinary effort in chemokine biology, extracellular vesicle biology, and apoptosis and mouse biology, with the potential to open new avenues of research in these areas that we are confident our work will ignite, which we think justifies the publication of the findings presented in this manuscript.
