# Supplementary material for: Chemokines act as phosphatidylserine-bound “find-me” signals in apoptotic cell clearance
Source: PLoS Biol. 2021 May 26;19(5):e3001259. doi: 10.1371/journal.pbio.3001259 (PMC8213124; doi:10.1371/journal.pbio.3001259)
Supplement: Attachment — Submitted filename: Response to Reviewers R3.pdf [file pbio.3001259.s018.pdf]

RE: PBIOLOGY-D-20-01298R3
"Chemokines act as phosphatidylserine-bound 'find-me' signals in apoptotic cell clearance"

REVIEWER 5

GENERAL COMMENTS

Firstly, I thank the authors for the efforts they have gone to in order to improve the manuscript in response the critical analysis by us and other reviewers. The rewording and new data shown has indeed improved clarity and provided more convincing support for their thesis of PS-binding by chemokines, and a putative role in phagocyte recruitment to clear apoptotic cells. Notably, the use of macrophage/monocytes to study migration; and the use of B18 binding and proteinase K to demonstrate the relative contribution of GAG versus PS-binding to chemokine retention on the surface of dying cells.

Response: We thank the Reviewer for these comments.

This reviewer remains skeptical about the real in vivo relevance of the chemokine-PS interaction in the context of apoptotic cell clearance. Nonetheless, I do agree that the experiments are for the most part thoroughly performed and the findings presented in this manuscript are of sufficient interest to justify publication. As the authors discuss, PS-binding by such a range of chemokines may indeed be pertinent to physiological/pathological processes outside the context of cell death and clearance. I have some small (but critical) suggestions for publication:

Response: Establishing PS-bound chemokine regulation of apoptotic cell clearance at the in vivo level is an important challenge that is beyond the scope of the present paper, as the Reviewer appears to acknowledge. Our paper sets the stage for such studies by the discovery of anionic phospholipid binding activity as a general property of many chemokines and by establishing that chemokine-PS binding occurs on apoptotic cells and blebs, that PS-binding chemokines associated with apoptotic blebs are functionally active, and that the phagocyte-recruiting activity of apoptotic bodies is mediated by vesicle-bound chemokines. We agree that chemokine-PS interactions may be relevant to multiple physiological/pathological/biological processes beyond apoptosis and dead cell clearance. As such, we think this paper will be of interest to a broad readership, such as that of PLoS Biology.

SPECIFIC COMMENTS/REQUESTS

Point 1: *Figure 4 "E) Quantification of the median fluorescence intensity (MFI) of the binding of the indicated biotinylated chemokines ... Bars represent mean ± SD MFI of duplicates. Results are from one experiment representative of 3 independent experiments. p values from multiple t tests with Holm-Sidak correction for multiple comparisons are indicated."*
>> Please confirm that statistics were performed on experimental replicates (greater than or equal to 3) and NOT technical duplicates. Showing S.D. for technical duplicates is incorrect.

Response: We have revised this figure to include all binding data generated for the different chemokines in 3 independent experiments (new Fig 4F). Data were normalized in relation to the binding of each chemokine to apoptotic thymocytes in the absence of competing annexin V (AnV) in each experiment, which was set at 100%. We have also included a new Fig 4E showing one representative FACS histogram for the binding of the different chemokines to apoptotic thymocytes.

Point 2: *Line 687 "this activity is vesicle-dependent" >>Use of centrifugation to deplete the vesicles (and presumably their associated chemokines) is a valid approach; but does not necessarily demonstrate that the chemotactic activity of these cytokines is dependent on vesicle integrity. Use of a surfactant would demonstrate that. Given the authors do not fully characterize their apoptotic vesicles/blebs beyond low SSC (ie to prove vesicle integrity) I would advocate removal of the reference to 'vesicle-dependent' activity.*

Response: As requested, the reference to "vesicle-dependent activity" has been removed from this line (now in Line 691).

Point 3: *Line 543: "BMDM, however, displayed a different pattern of responsiveness to thymus-derived SN and ApoBD…" and Line 546: "These results reinforce the role of ApoBD as strong phagocyte-recruiting factors and indicate that unlike in the case of monocytes, functional levels of macrophage-recruiting agents …"*
>> The authors should mention briefly the considerations of using mouse-derived chemokines to induce cellular responses mediated by human receptors. Moreover, the difference in receptor expression (and their cross species ligand recognition) on the monocyte/macrophages used may explain the differential responsiveness to the ApoBD derived from PBS or DEX injected mice?

Response: We agree with the Reviewer. As requested, these notions have been included as a possible explanation for the different chemotactic response pattern observed between mouse BMDM and human monocytes (See Lines 556-560).

Point 4: *>>Although an admirable attempt, the use of apyrase in an ex vivo context does not seem to be an entirely pertinent experiment. That is, ATP release is a potent chemoattractant acting over a short distance from dying cell target to phagocyte. Yet in a context in which the ApoBD have been isolated from the target, one would intuit that the relative contribution of ATP to their chemotactic effect is, of course, negligible.*

Response: We agree that the inability of apyrase to inhibit ApoBD-induced phagocyte migration does not address the well-established find-me signal activity of ATP in other contexts. We have included a new statement to make this clearer (see Line 581-583). However, we think that apyrase does serve a useful role in our experiments simply as a protein specificity control of a recombinant protein that does not inhibit ApoBD-mediated migration.

Point 5: *Figure 6E could be moved to supplemental data.*

Response: As suggested, Fig 6E has been moved to a new supplementary S10 Fig.

Point 6: *>>Right column in figure 3A - label the title (eg 'binding to liposomes')*

Response: As requested, titles have been added to each graph column of Fig 3A and the legend to improve clarity.
